# Structure and Expression Analysis of Sucrose Phosphate Synthase, Sucrose Synthase and Invertase Gene Families in *Solanum lycopersicum*

**DOI:** 10.3390/ijms22094698

**Published:** 2021-04-29

**Authors:** Yaoke Duan, Lan Yang, Haijia Zhu, Jie Zhou, Hao Sun, Haijun Gong

**Affiliations:** 1 Shaanxi Engineering Research Center for Vegetables/College of Horticulture, Northwest A & F University, Yangling 712100, China; duyk@nwafu.edu.cn (Y.D.); lanyang666@nwafu.edu.cn (L.Y.); 060315zhj@nwafu.edu.cn (H.Z.); xb98@nwafu.edu.cn (J.Z.); 2 Henan Key Laboratory of Ion-Beam Bioengineering, College of Agricultural Sciences, Zhengzhou University, Zhengzhou 450000, China

**Keywords:** *Solanum lycopersicum*, sucrose phosphate synthase, sucrose synthase, invertase, structure characteristics, gene expression

## Abstract

Sucrose phosphate synthase (SPS), sucrose synthase (SUS) and invertase (INV) are all encoded by multigene families. In tomato (*Solanum lycopersicum*), a comprehensive analysis of structure characteristics of these family genes is still lacking, and the functions of individual isoforms of these families are mostly unclear under stress. Here, the structure characteristics of the three families in tomato were analyzed; moreover, as a first step toward understanding the functions of isoforms of these proteins under stress, the tissue expression pattern and stress response of these genes were also investigated. The results showed that four *SPS* genes, six *SUS* genes and nineteen *INV* genes were identified in tomato. The subfamily differentiation of *SlSPS* and *SlSUS* might have completed before the split of monocotyledons and dicotyledons. The conserved motifs were mostly consistent within each protein family/subfamily. These genes demonstrated differential expressions among family members and tissues, and in response to polyethylene glycerol, NaCl, H_2_O_2_, abscisic acid or salicylic acid treatment. Our results suggest that each isoform of these families may have different functions in different tissues and under environmental stimuli. *SlSPS1*, *SlSPS3*, *SlSUS1*, *SlSUS3*, *SlSUS4*, *SlINVAN5* and *SlINVAN7* demonstrated consistent expression responses and may be the major genes responding to exogenous stimuli.

## 1. Introduction

Plants are autotrophic organisms that can fix carbon dioxide and produce carbohydrates through photosynthesis. Sucrose is the main end product of photosynthesis in higher plants, and it is transported from the source leaves to sink organs [1]. Sucrose plays an important role in plant growth and development. On the one hand, it provides energy and structural components of plants; on the other hand, sucrose and its hydrolysis products may function as signaling molecules, which regulate the expression of genes involved in important physiological processes [2,3]. Sucrose also functions in osmotic adjustment [3] and thus plays an important role in stress adaption.

The key enzyme responsible for sucrose synthesis is sucrose phosphate synthase (SPS), which catalyzes the formation of 6-phosphate sucrose from UDP-glucose and 6-phosphate fructose [1]. The main enzymes involved in sucrose catabolism are invertase (INV) and sucrose synthase (SUS)—invertase catalyzes the degradation of sucrose into glucose and fructose, whereas SUS converts sucrose into UDP-glucose and fructose [4]. Invertases can be classified as acid invertase and alkaline/neutral (A/N) invertase based on their optimum pH [5]. Acid invertases are usually localized in the cell wall or vacuole, and alkaline/neutral invertases are localized in the cytosol, mitochondria or plastid [2]. SUS proteins are mainly localized in the cytosol or plasma membrane, and some are localized in the vacuole, cell wall or mitochondria [1].

SPS, SUS and INV are all encoded by multigene families and have been characterized in some plant species. SPS is encoded by a small family. For example, in the model plants Arabidopsis (*Arabidopsis thaliana*) and rice (*Oryza sativa*), there are four and five *SPS* members, respectively [6,7]. Maize (*Zea mays*) and apple (*Malus domestica*) both have six members of *SPS* genes [8,9], whereas *Amborella trichopoda* only has two *SPS* genes [10]. According to phylogenetic analysis, *SPS* genes are categorized into four families (A, B, C and D), and family D only exists in some monocots [6,8]. The member number of *SUS* genes varies greatly among plant species. In *Amborella trichopoda*, only two *SUS* genes have been identified [11]. There are six, eight, twelve and fourteen *SUS* genes in Arabidopsis, carrot (*Daucus carota*), soybean (*Glycine max*) and tobacco (*Nicotiana tabacum*), respectively [11,12]. In Chinese pear, it is reported that there were thirty *SUS* genes [13]. *SUS* genes can be classified into three subfamilies—SUS I, SUS II and SUS III [1]. The genes encoding invertase have been identified in some plant species. For instance, in Arabidopsis, eight acid invertase genes and nine A/N invertase genes have been identified [14]. There are eight acid invertase genes and six A/N invertase genes in sugarcane [15], and these corresponding numbers in soybean are nineteen and thirteen, respectively [16]. In tomato (*Solanum lycopersicum*), an important vegetable crop, four *SPS* genes [17], six *SUS* genes [11], eleven acid invertase genes [5] and eight alkaline/neutral invertase encoding genes [2] have also been identified. However, the structure characteristics of the genes have not been comprehensively analyzed, except those of one type of invertase—A/N invertase [2].

Up to date, studies on the functions of genes encoding SPS, SUS and INV have mostly focused on growth, development and fruit quality, especially the former two aspects. Bahaji et al. suggested that SPS is essential for Arabidopsis viability by using double and triple mutants of *SPSA1*, *SPSA2*, *SPSB* and *SPSC* [18]. In sugarcane, *SoSPS1* overexpression increased the plant height and stalk number of some transgenic lines [19]. Heterologous expression of a spinach *SPS* gene in cotton improved the fiber quality [20], while the expression of the Arabidopsis family A *SPS* gene in tobacco increased the fiber length [21]. Park et al. reported that expression of *Arabidopsis SPS* gene in poplar not only increased the length of xylem fibers, but also delayed the onset of senescence and advanced the bud flush [22]. In addition, *SPS* genes may also be involved in the regulation of flowering time, flower number, pollen germination and fruit development [23,24,25]. SUS is considered as a biochemical marker of sink strength [11], and it plays roles in the development of vascular tissues and shoot apical meristem. *SUS* genes are highly expressed in plant vascular tissues and are involved in the synthesis of both cellulose and callose [1]. Barratt et al. observed that Arabidopsis double mutant *sus5*/*sus6* had reduced callose levels in the sieve plates of phloem [26]. Wei et al. found that overexpression of poplar xylem *SUS2* in tobacco increased the cellulose content and the thickness of the xylem cell wall [27]. In tomato, Goren et al. reported that suppression of *SUS* genes did not affect the carbohydrate levels in the fruits, but affected auxin signaling and the leaf morphology, indicating the possible roles of SUS in regulating early leaf development [28]. SUS may also play important roles in mutualism with symbiotic organisms and fruit ripening [29,30]. Invertase is also involved in the regulation of plant growth and development. Knockout mutation in A/N-InvC caused a reduction in shoot growth and oxygen consumption, suggesting that the invertase played an important role in the respiratory process in mitochondria [31]. In rice, the mutation of *CYT-INV1* inhibited the root growth and delayed flowering [32]. Zanor et al. found that silencing the expression of *LIN5*, which encodes a cell wall invertase in tomato, changed the flower and fruit morphology, increased the petal and sepal numbers of flowers, and decreased pollen viability and seed size, further confirming the important role of invertase in reproductive growth of plants [33].

In plants, although the roles of some *SPS*, *SUS* and *INV* genes in growth and development have been investigated, responses of these genes to environmental stresses are largely unknown, and information on their roles under stress conditions is very limited. Takehara et al. reported that high expression of *SUS3* in brown rice was associated with its heat tolerance [34]. Wang et al. found that inhibition of *CsSUS3* expression decreased the tolerance of cucumber to hypoxic stress [35]. Qian et al. found that overexpression of tea *CsINV*5 enhanced the cold tolerance of Arabidopsis [36]. In tomato, Liu et al. and Xu et al. reported that manipulating the expression of cell wall invertase inhibitor gene could regulate fruit setting under heat stress and chilling tolerance [37,38]. Since individual members of *SPS*, *SUS* and *INV* family genes in plants have distinct tissue expression patterns and have been found to demonstrate different functions in growth and development [16,26], the functions of the individual isoforms of these enzymes under stress conditions may be different. In this study, comprehensive analyses on the structural characteristics of genes encoding SPS, SUS and INV in tomato were conducted. As a first step toward understanding the functions of isoforms of these enzymes under stress conditions, the tissue expression pattern and the expression response of these genes to different environmental stimuli were also investigated. The study may help us better understand the gene structure characteristics of the main sucrose metabolism related enzymes, and provide a basis for investigating the roles of individual isoforms of each enzyme in growth, development and stress tolerance of tomato plants.

## 2. Results

### 2.1. Identification of SPS, SUS and INV Genes in Tomato Genome

To identify the *SPS* genes in tomato, iterative protein BLAST analysis was performed using previously identified Arabidopsis SPS sequences as the queries. The candidate *Solanum lycopersicum* *SPS* (*SlSPS*) genes were further screened by BLASTp in Swiss-Prot database and Batch-CDD tests. As a result, four *SlSPS* genes were identified. The genes were renamed based on their chromosome numbering, and the basic characteristics of these genes and the corresponding proteins were analyzed (Table 1). These *SlSPS* genes had similar CDS lengths, and the corresponding proteins shared similar amino acid sequence lengths, molecular weights and isoelectric points. The online tool Kinasephos was used to predict the phosphorylation sites, and the results showed that the SPS proteins have 15–24 putative phosphorylation sites. All the SPS proteins were predicted to be localized at the plasma membrane.

Using the same methods, including two rounds of BLAST and screening by structure characteristics, six *SlSUS* genes were identified in the tomato genome (Table 1). The CDS of *SlSUS* genes had 2412–2676 bp, and the length of corresponding polypeptides was 803–891 Aa. The isoelectric point of these SUS proteins was 5.87–6.94, and the molecular weight range was 91.6–100.7 KDa. The six SUS proteins were all predicted to be localized at the plasma membrane. The SlSUS contained 9–20 phosphorylation sites, with SUS6 possessing the highest number.

Nineteen *INV* genes were identified in the tomato genome, including 11 acid invertase genes and eight A/N invertase genes (Table 1). According to the predicted subcellular localization of the corresponding proteins, the acid invertase genes can be further divided into cell wall invertase genes (*INVCW1–9*) and vacuolar invertase genes (*INVVR1–2*). We named these genes according to the property and subcellular localization of the corresponding proteins, and numbered them based on their localizations on chromosomes. The CDS length of the *INVCW* genes and the corresponding amino acid sequence and molecular weight of the proteins were similar, except those of *INVCW2* and *INVCW6*, which were slightly shorter or lower. The isoelectric point and number of putative phosphorylation sites varied among the INVCWs, ranging from 4.96 to 9.23, and from 5 to 14, respectively. The two vacuolar invertase genes (*INVVR1–2*) had similar CDS lengths, and the isoelectric points and molecular weights of the corresponding proteins were also similar. The length of *SlINVAN* CDS and amino acid sequence varied among family members, with ranges of 1656–2019 bp and 551–672 Aa, respectively. Subcellular localization prediction of these INVAN proteins showed that two (INVAN1/2) were localized in the mitochondria, two (INVAN6/8) in the chloroplast, and others in the cytoplasm.

### 2.2. Phylogenetic Analysis of the SPS, SUS and INV Families

**Figure 1:**
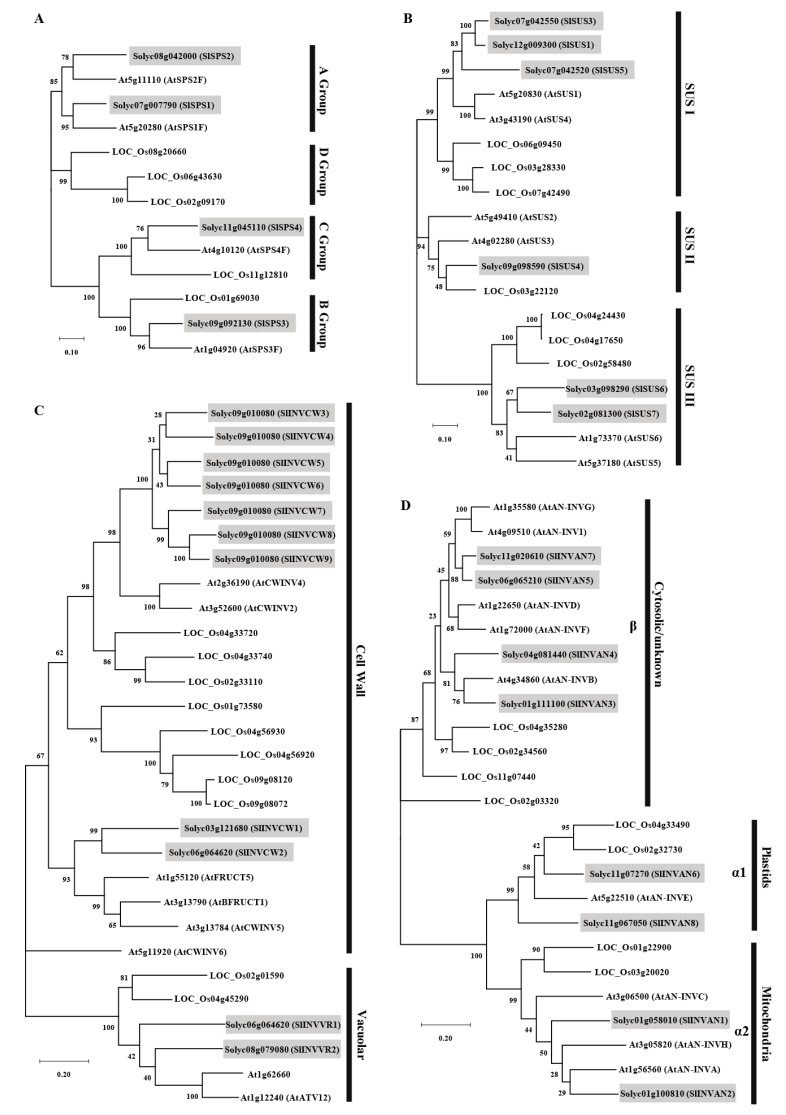
Phylogenetic analysis of SPS (**A**), SUS (**B**), acid INV proteins (**C**) and alkaline/neutral INV proteins (**D**). Trees were built using amino acid sequences from *Arabidopsis thaliana*, tomato (*Solanum lycopersicum*) and rice (*Oryza sativa*). The phylogenetic tree was constructed using the maximum likelihood method with MEGA-X.

To analyze the phylogenetic relationship of SPSs, SUSs or INVs from tomato with those from Arabidopsis and rice, which are, respectively, dicotyledonous and monocotyledonous model plants, an unrooted maximum likelihood tree was constructed using the MEGA software. As shown in Figure 1A, the SlSPS had a closer relationship with AtSPS than OsSPS. SPS from these three plant species could be divided into four distinct families: A, B, C and D. SlSPS1 and SlSPS2 were grouped into family A, SlSPS3 was grouped into family B, and SlSPS4 belonged to family C. No SPS from tomato or Arabidopsis was grouped into family D (Figure 1A).

The evolutionary relationship of SUS proteins in tomato, Arabidopsis and rice was analyzed using the amino acid sequences of 19 SUSs from these species (Figure 1B). These SUS proteins can be divided into three groups: SUS I, SUS II and SUS III. SlSUS1, SlSUS3 and SlSUS5 belonged to the SUS I group and were clustered together, and they were close to AtSUS1 and AtSUS4 in Arabidopsis. SlSUS4 belonged to SUS II, and it was closer to a SUS (LOC_Os03g22120) in rice, rather than to that in Arabidopsis. SlSUS6 and SlSUS7 belonged to SUS III, and they were close to AtSUS5 and AtSUS6.

To explore the phylogenetic relationship of acid invertase protein in tomato, Arabidopsis and rice, we constructed a phylogenetic tree based on the amino acid sequences of 11 SlINVs, 8 AtINVs and 10 OsINVs (Figure 1C). The acid invertases were grouped into two clades: 23 INVCWs were predicted to be localized in the cell wall and six INVVRs in the vacuole. SlINVCW3–9 and SlINVCW1–2 were clustered together, respectively. All the acid INVs in tomato were closer to those in Arabidopsis, rather than to those in rice. The phylogenetic relationship of A/N invertases from tomato, Arabidopsis and rice was also analyzed (Figure 1D). According to the phylogenetic tree, these invertases can be classified into two groups—α and β. SlINVAN3, 4, 5, and 7 belonged to the β group, and the remaining four belonged to the α group. The α group could be further divided into α1 and α2 subgroups according to their subcellular localization. In tomato, SlINVAN6 and 8 belonged to the α1 group, whereas SlINVAN1 and 2 belonged to the α2 group.

### 2.3. Gene Structure and Conserved Protein Motif Analysis of SPS, SUS and INV Gene Families

**Figure 2:**
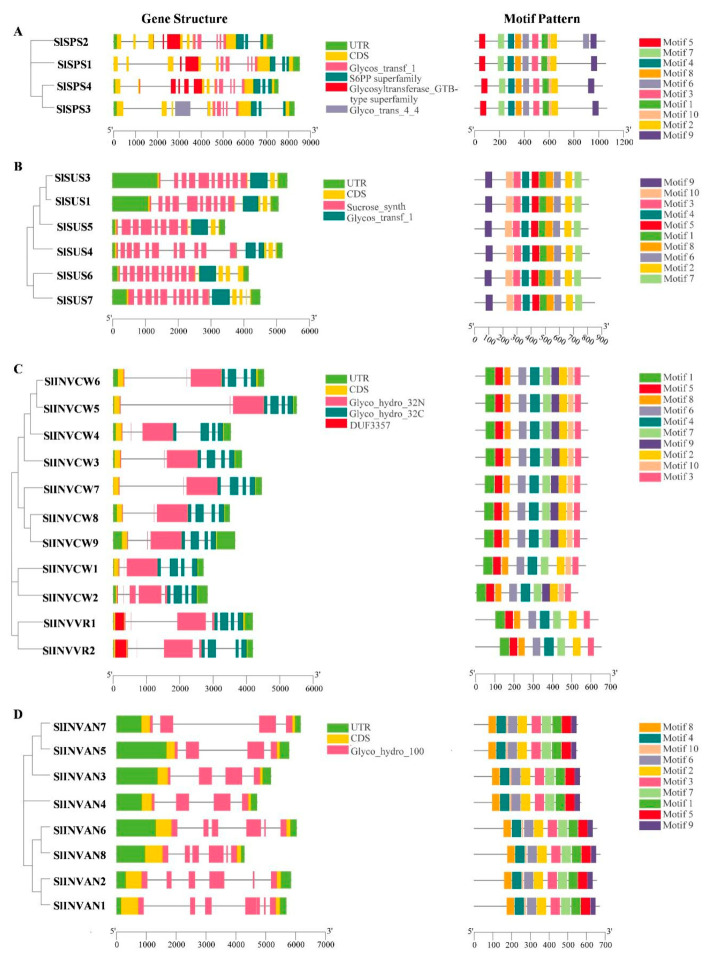
Gene structure and conserved motif analysis of SPS (**A**), SUS (**B**), acid INV (**C**) and alkaline/neutral INV (**D**) in tomato. In the gene structure, the filled boxes and lines represent exons and introns, respectively. The green and yellow boxes, respectively, represent UTR sequence and CDS. In the motif map, different conserved motifs were shown in different colors.

The gene structure display server (GSDS) online program was used to analyze the intron-exon structure. Analysis on the conserved domain structure was based on the online program of NCBI BATCH CD-search tool (https://www.ncbi.nlm.nih.gov/Structure/bwrpsb/bwrpsb.cgi, accessed on 12 April 2020). The MEME search tool was employed to predict the conserved protein motifs. The results showed that the *SlSPS* genes had 12–14 exons and 11–13 introns (Figure S1). Glucosyl transferase Glycos-transf-1 domain (pfam00534) and Glyco-trans-4-4 domain (pfam13579) belong to the Glucosyl transferase GTB-type-superfamily (cl10013). All the SlSPS members had Glycos-transf-1 and S6PP domains (cl37722) (Figure S2), and the Glyco-trans-4-4 domain only existed in SlSPS3 (Figure 2A). The Glycos-transf-1 domain is related to the transfer of glucosyl, and S6PP domains (SPP-like domain) may be the site of SPS binding SPP [46,47]. Ten conserved motifs were predicted in each SPS protein, and most of the motifs were repeated once, except motif 6, which was repeated twice only in SlSPS2 (Figure 2A and Figure S3A). Taken the analysis results of the motif and domain together, it was found that motifs 3, 1, 10 and 2 belonged to the Glycos-transf-1 domain, while motif 9 belonged to the S6PP domain. In SPS1 and SPS2, motifs 7, 4, 8 and 6 belonged to the GTB-type-superfamily; in SPS4, motifs 5, 7, 4 and 8 belonged to the GTB-type-superfamily; and in SPS3, motifs 7, 4, 8 and 6 belonged to the Glyco-trans-4-4 domain.

The six *SlSUS* genes contained 11–15 exons (Figure S1). These genes shared two typical domains: sucrose synthase domain (pfam00862) and glucosyl transferase domain (pfam00534) (Figure 2B and Figure S4). Ten conserved motifs existed in these SUS proteins and each motif was repeated once (Figure 2B and Figure S3B). The motifs 9, 10, 3, 4, 5 and 1 belonged to the sucrose synthase domain, whereas motifs 8, 6, 2 and 7 belonged to glucosyl-transferase domain (Figure 2B).

To explore the structure characteristics of acid invertase genes in tomato, the intron-exon structure and conserved motifs were analyzed. Except *INVCW2*, which had eight exons, the other *INVCW* genes all had six exons (Figure S1). Both *INVVR1* and *INVVR2* contained seven exons (Figure S1). It is interesting to note that the second exon of all acid invertase genes contained only nine nucleotide acids (Figure S1). The N-terminal of acid invertase is glycosylated and it belongs to glycohydrolase family 32 (Glycoside Hydrolase Family 32, GH32) [48]. The cysteine catalytic MWECP/V and β-furosidase motif NDPNG/A were conservative in all the tomato acid invertase sequences (Figure S5). Both INVCWs and INVVRs in tomato had the Glyco-hydro-32N and Glyco-hydro-32C domains, and the DUF3357 domain was only present in INVVRs (Figure 2C). Motif analysis showed that SlINVCWs contained motifs 1–10 except INVCW1, where motif 9 was lacking (Figure 2C and Figure S3C). Another interesting finding is that some motifs were only conserved in SlINVCWs. For instance, motif 9 and motif 10 were present in SlINVCWs but missing in SlINVVR proteins. Motifs 1, 5, 8, 6, 4 and 7 belonged to the Glyco-hydro-32N domain, while motifs 7, 9, 2, 10 and 3 belonged to Glyco-hydro-32C domain (Figure 2C).

There were eight A/N invertase genes in tomato (Table 1). Among these, *SlINVAN1*, *2*, *6* and *8*, which encoded α group invertases, contained 6–7 exons, whereas *SlINVAN3*, *4*, *5* and *7*, which encoded β group invertases, had four exons (Figure S1). A/N invertases are nonglycosylated proteins, belonging to the glycosyl hydrolase family 100 (GH100) [48]. The Glyco-hydro-100 domain was observed in all the *SlINVAN* genes (Figure 2D). The eight SlINVAN proteins shared 10 conserved motifs, which had similar arrangement (Figure 2D and Figure S3D). It is noted that the amino acid sequence before the first motif (motif 8) was longer in the α group invertases than that in the β group invertases (Figure 2D).

### 2.4. Chromosome Distribution, Synteny Analysis of SPS, SUS and INV Genes

Chromosome distribution of *SPS*, *SUS* and *INV* gene families was analyzed base on the physical location of the GCF_000188115.4_SL3.0_genomic database from the NCBI website. Gene duplication plays an important role in the amplification of gene family and subsequent evolution, with segmental and tandem duplications being the main causes of gene family expansion in plants [49]. Duplication events of these genes were analyzed using the MCScanX software. The syntenic relationship of *SPS*, *SUS* and *INV* genes in different plant species was analyzed using the Dual Synteny Plotter software.

**Figure 3:**
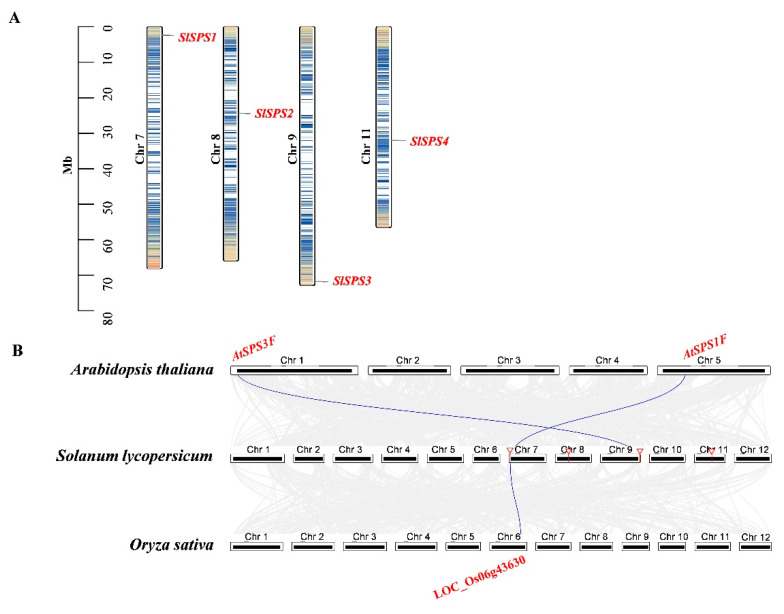
Chromosome localization of tomato *SPS* genes (**A**) and synteny analysis of *SPS* genes from tomato, Arabidopsis and rice (**B**). Chr, chromosome.

Our results showed that the *SlSPS* genes were distributed on four chromosomes—*SPS1*, *SPS2*, *SPS3* and *SPS4* on chromosomes 7, 8, 9 and 11, respectively—and no tandem duplication or segmental duplication was detected (Figure 3A). Synteny analysis between *SPSs* in tomato and those in both Arabidopsis and rice indicated that *AtSPS1F-SlSPS1*, *SlSPS1*-LOC_Os06g43630 and *AtSPS3F*-*SlSPS3* were, respectively, identified as syntenic gene pairs (Figure 3B).

**Figure 4:**
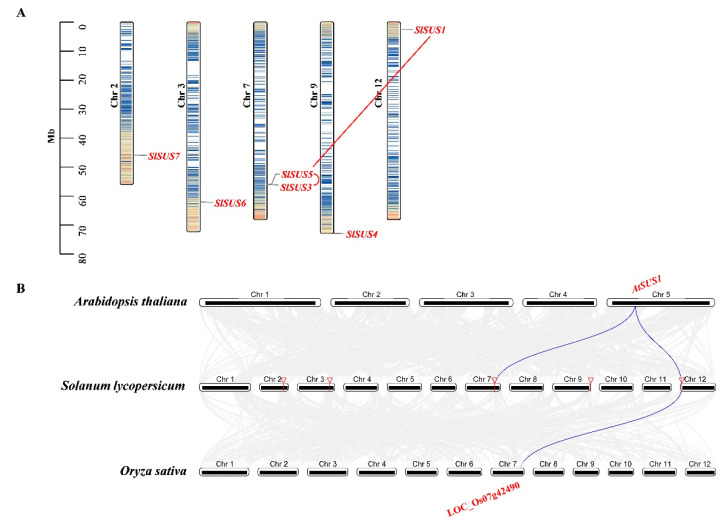
Chromosome localization of tomato *SUS* genes (**A**) and synteny analysis of *SUS* genes from tomato (**B**), Arabidopsis and rice. The red arc line indicates tandem duplicated gene pair, and the red straight line indicates segmental duplicated gene pair. Chr, chromosome.

The six tomato *SUS* genes were distributed on five chromosomes (chromosomes 2, 3, 7, 9 and 12) (Figure 4A). Both *SlSUS3* and *SlSUS5* were located on chromosome 7, and they were identified as tandem duplication genes (Figure 4A). *SlSUS1* and *SlSUS5* were identified as segmental duplication gene pairs. Synteny analysis showed that there were three syntenic gene pairs: *AtSUS1*-*SlSUS1*, *AtSUS1*-*SlSUS5* and *SlSUS1*- LOC_Os07g42490.

**Figure 5:**
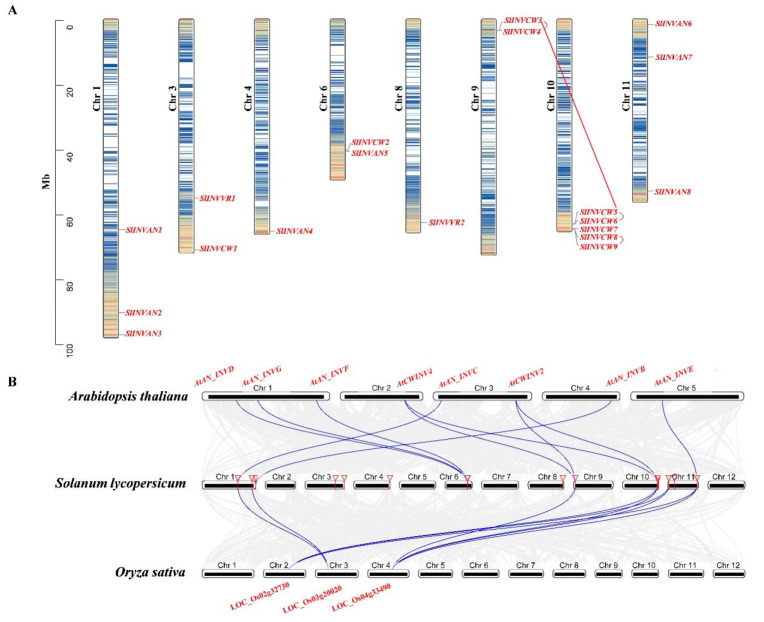
Chromosome localization of tomato *INV* genes (**A**) and synteny analysis of *INV* genes from tomato, Arabidopsis and rice (**B**). The red arc lines indicate tandem duplicated gene pairs, and the red straight line indicates segmental duplicated gene pair. Chr, chromosome.

There were 19 *INV* genes in the tomato genome, including 11 acid *INV* and eight A/N *INV* genes; these genes were unevenly distributed on eight chromosomes (Figure 5A). For instance, on chromosome 10, there were five *INV* genes, whereas there was only one *INV* gene on chromosomes 4 and 8, respectively (Figure 5A). Gene duplication analysis showed that there were three tandem duplication pairs (*INVCW3*/*INVCW4*, *INVCW5/INVCW6* and *INVCW8*/*INVCW9*) and two segmental duplication gene pairs (*INVCW3*/*INVCW5*, *INVAN6*/*INVAN8*). To further determine the origin and evolution dynamics of tomato *INV* genes, we investigated the syntenic relationship between *INV* genes in tomato and those in *Arabidopsis thaliana* and rice. The result showed that 10 syntenic gene pairs between tomato and Arabidopsis and nine pairs between tomato and rice were identified, and they were listed as follows: *AtAN_INVC*-*SlINVAN1*-LOC_Os03g20020, *AtAN_INVB*-*SlINVAN3*, *AtAN_INVD*-*SlINVAN5*, *AtAN_INVF*-*SlINVAN5*, *AtAN_INVG*-*SlINVAN5*, *AtCWINV2*-*SlINVCW3*-LOC_Os04g33720, *AtCWINV4*-*SlINVCW3*-LOC_Os04g33720, *AtCWINV2* -*SlINVCW5*-LOC_Os04g33720, *AtCWINV2*-*SlINVCW5*-LOC_Os02g33110, *AtCWINV4*-*SlINVCW5*-LOC_Os04g33720, *AtCWINV4*-*SlINVCW5*- /LOC_Os02g33110, *AtAN_INVE*-*SlINVAN8*-LOC_Os02g32730, *AtAN_INVE*-*SlINVAN8*-LOC_Os04g33490, *SlINVAN6*-LOC_Os02g32730. *SlINVAN6*-LOC_Os04g33490. The *INV* genes in tomato which showed syntenic relationships with those in Arabidopsis or rice were mainly located at chromosomes 1, 6, 9, 10 and 11.

### 2.5. Tissue Expression Profile of SUS, INV and SPS in Tomato

**Figure 6:**
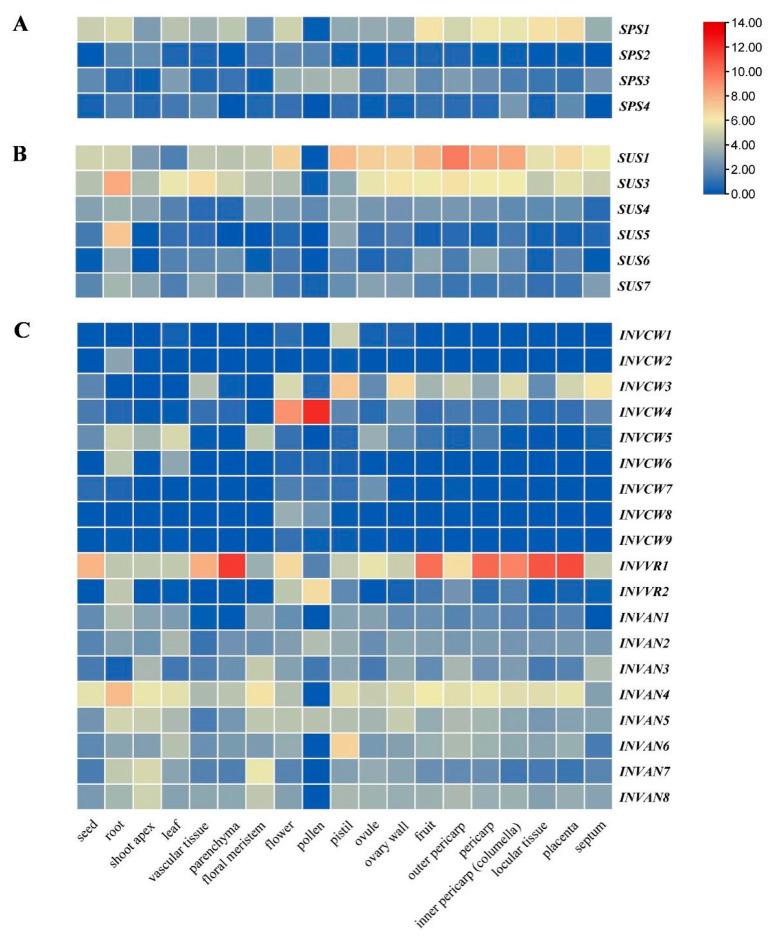
Tissue expression pattern of *SPS*, *SUS* and *INV* genes in tomato. The expression values were retrieved from large scale RNA-seq with Genevestigator. (**A**) *SPS* expression; (**B**) *SUS* expression; (**C**) *INV* expression.

Both bioinformatic and experimental approaches were employed to determine the tissue expression profiles of *SPS*, *SUS* and *INV* genes in tomato. The expression data collected from public RNA-seq repositories demonstrated that each family gene showed differential tissue expression patterns (Figure 6). For instance, the expression of *SPS1* was higher than that of any other *SPS* in the fruit, columella, locular tissue, pericarp, placenta and septum (Figure 6A). In the *SUS* family, compared with other members, *SUS1* and *SUS3* were both highly expressed in the fruit, columella, outer pericarp, ovary wall, ovule and pericarp (Figure 6B). The expression of *SUS5* was much higher in roots than any other tissues (Figure 6B). Among the *INV* family genes, by and largely, *INVVR1* and *INVAN4* (especially the former) were highly expressed in most tissues (Figure 6C). In addition, *INVCW4* appeared to be specifically expressed in the flower and pollen (Figure 6C).

**Figure 7:**
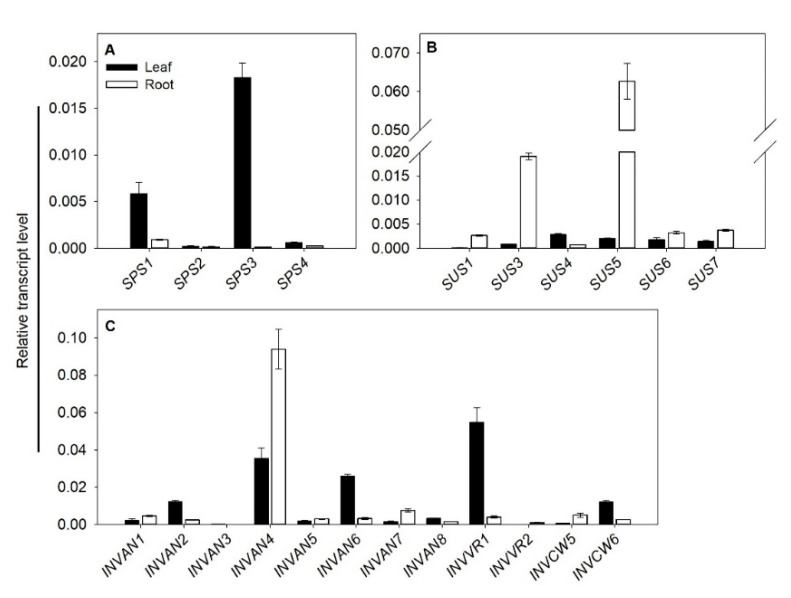
Expression of *SPS*, *SUS* and *INV* genes in leaves and roots of tomato. Expression was monitored using quantitative RT-PCR at three-leaf stage. (**A**) *SPS* expression; (**B**) *SUS* expression; (**C**) *INV* expression.

A quantitative RT-PCR technique was also introduced to analyze the expression of *SUS*, *INV* and *SPS* genes in the leaf and root of tomato. The results showed that all the SPS genes in tomato were mainly expressed in the leaf, and both *SPS1* and *SPS3* (especially the latter) demonstrated much higher expression than the other two *SPS* genes (Figure 7A). Except *SUS4*, most *SUS* genes showed higher expression in the root than the leaf, with *SUS3* and *SUS5* being the top two highly expressed *SUS* genes (Figure 7B). We also analyzed the tissue expression of all mined *SlINVAN* and *SlINVVR* genes, as well as *SlINVCW5* and *SlINVCW6*—two highly expressed *SlINVCW* genes observed in the collected public data (Figure 6C and Figure 7C). Among the *SlINVANs*, *SlINVAN4* was highly expressed in both leaves and roots (Figure 7C). *SlINVAN6* and *SlINVAN7* also had relatively high expression in the leaf and root, respectively (Figure 7C). *SlINVVR1* demonstrated high expression in the leaf. In brief, by and large, these results are consistent with the public RNA-seq data.

### 2.6. Expression of SPS, SUS and INV Genes under Simulated Stress Conditions in Tomato

**Figure 8:**
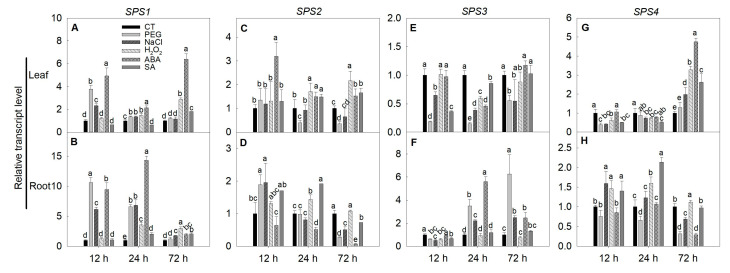
Changes in the expression of *SPS* family genes under different exogenous stimuli conditions. The three-leaf age seedlings were treated with 20% (*w*/*w*) polyethylene glycerol-6000 (PEG-6000), 150 mM NaCl, 1.5 mM H_2_O_2_, 50 μM abscisic acid (ABA) or 200 μM salicylic acid (SA) for 12 to 72 h, after which the leaves and roots were collected for gene expression analysis using quantitative RT-PCR. (**A**) *SPS1* expression in leaves; (**B**) *SPS1* expression in roots; (**C**) *SPS2* expression in leaves; (**D**) *SPS2* expression in roots; (**E**) *SPS3* expression in leaves; (**F**) *SPS3* expression in roots; (**G**) *SPS4* expression in leaves; (**H**) *SPS4* expression in roots. Actin, TIP41 and 17sRNA were used as internal controls. Data are means ± SD (*n* = 3). Different letters above bars indicate a significant difference at *p* < 0.05. CT, control.

PEG and NaCl are usually used to simulate drought and salinity stress, respectively. H_2_O_2_, one of the main reactive oxygen species and signaling molecules, usually accumulates under stress conditions. ABA and SA are also signaling molecules and respond to environmental stresses. In this study, PEG, NaCl, H_2_O_2_, ABA, and SA treatments were applied to simulate various stress conditions. The results showed that the expression of *SPS1* was largely induced by all the treatments, especially in the root (Figure 8A,B). *SPS2* expression was increased in the leaf but decreased in the root by ABA treatment (Figure 8C,D). PEG and NaCl treatments for 72 h decreased *SPS2* expression in the leaf and root (Figure 8C,D). The expression of *SPS3* was decreased in the leaf by all the treatment at various time points, while in the root, the expression was decreased by PEG and NaCl treatments at 12 h, but it was increased later (Figure 8E,F). ABA treatment also stimulated *SPS3* expression in the root at later stress periods (Figure 8F). *SPS4* expression in the leaf was slightly decreased at 12 and 24 h by all the five treatments, but it was differentially increased at 72 h, while in the root, the expression was increased by H_2_O_2_ and SA treatments at 12 and 24 h, but decreased by PEG and ABA treatments at a later period.

**Figure 9:**
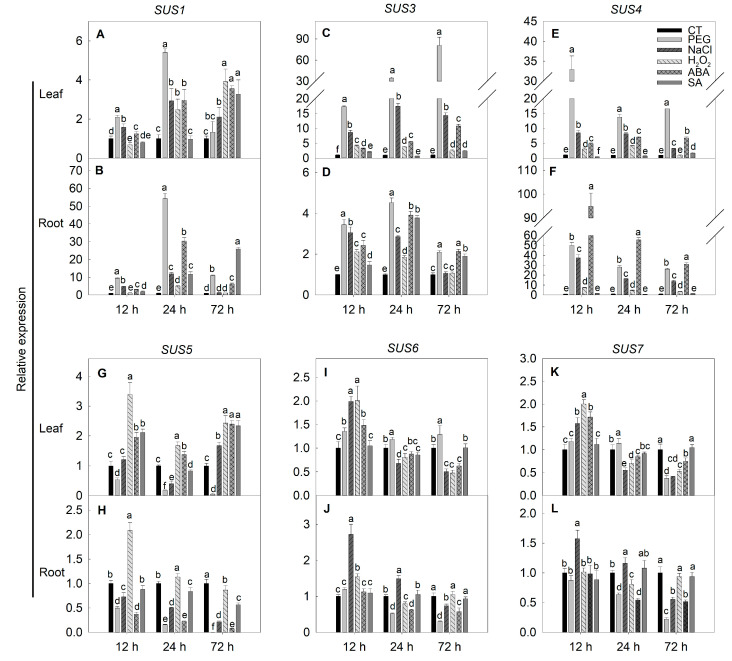
Changes in the expression of *SUS* family genes under different exogenous stimuli conditions. The three-leaf age seedlings were treated with 20% (*w*/*w*) polyethylene glycerol-6000 (PEG-6000), 150 mM NaCl, 1.5 mM H_2_O_2_, 50 μM abscisic acid (ABA) or 200 μM salicylic acid (SA) for 12 to 72 h, after which the leaves and roots were collected for gene expression analysis using quantitative RT-PCR. (**A**) *SUS1* expression in leaves; (**B**) *SUS1* expression in roots; (**C**) *SUS3* expression in leaves; (**D**) *SUS3* expression in roots; (**E**) *SUS4* expression in leaves; (**F**) *SUS4* expression in roots; (**G**) *SUS5* expression in leaves; (**H**) *SUS5* expression in roots; (**I**) *SUS6* expression in leaves; (**J**) *SUS6* expression in roots; (**K**) *SUS7* expression in leaves; (**L**) *SUS7* expression in roots. Actin, TIP41 and 17sRNA were used as internal controls. Data are means ± SD (*n* = 3). Different letters above bars indicate a significant difference at *p* < 0.05. CT, control.

The *SUS* family members also demonstrated differential expression responses to the chemical treatments. The expression of *SUS1*, *SUS3* and *SUS4* in the leaf and root was largely increased in response to the treatment of five chemicals, except at 12 h, when *SUS1* and *SUS4* expression was decreased by H_2_O_2_ and SA treatments, respectively (Figure 9A–F). Under PEG stress, the expression of *SUS5* was significantly decreased in the leaf and root (Figure 9G,H). In the leaf, *SUS5* expression was increased by H_2_O_2_ and SA treatments, whereas the expression was decreased by NaCl and ABA treatments in the root (Figure 9G,H). The expression of *SUS6* and *SUS7* in the leaf and root was stimulated by NaCl treatment at the early stress period, but they were decreased at the later period (Figure 9I,L). The expression of these two genes was inhibited at a late period of PEG treatment (Figure 9I–L). Neither the expression of *SUS6* nor that of *SUS7* was affected by SA treatment (Figure 9I–L).

**Figure 10:**
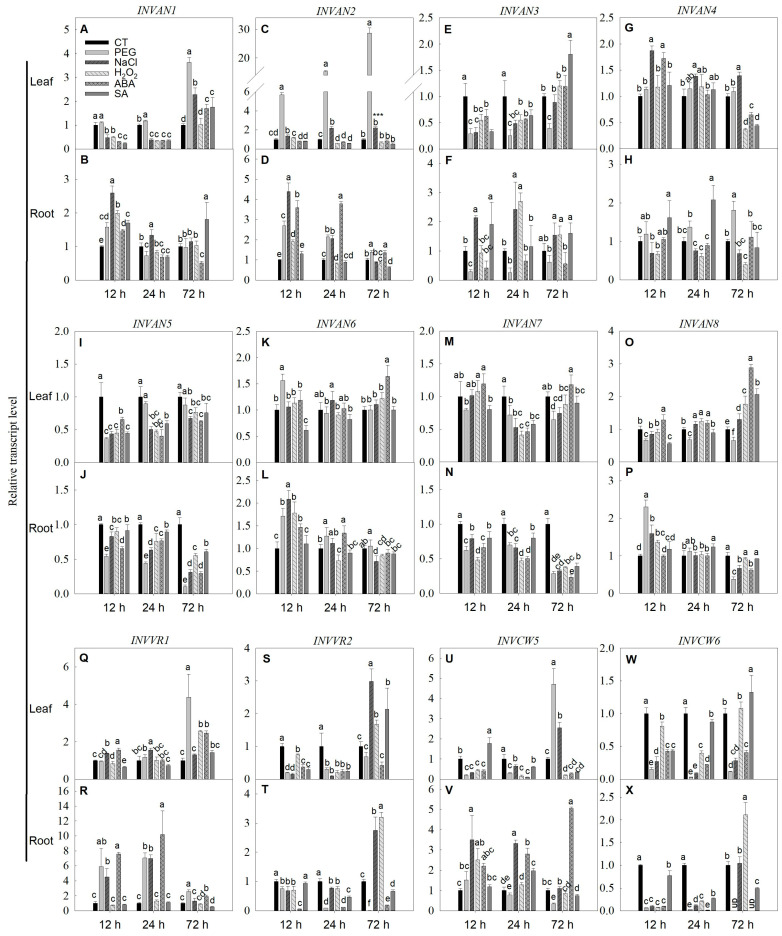
Changes in the expression of *INV* family genes under different exogenous stimuli conditions. The plants were treated with 20%(*w*/*w*) polyethylene glycerol-6000 (PEG-6000), 150 mM NaCl, 1.5 mM H_2_O_2_, 50 μM abscisic acid (ABA) or 200 μM salicylic acid (SA) for 12 to 72 h, after which the leaves and roots were collected for gene expression analysis using quantitative RT-PCR. (**A**) *INVAN1* expression in leaves; (**B**) *INVAN1* expression in roots; (**C**) *INVAN2* expression in leaves; (**D**) *INVAN2* expression in roots; (**E**) *INVAN3* expression in leaves; (**F**) *INVAN3* expression in roots; (**G**) *INVAN4* expression in leaves; (**H**) *INVAN4* expression in roots; (**I**) *INVAN5* expression in leaves; (**J**) *INVAN5* expression in roots; (**K**) *INVAN6* expression in leaves; (**L**) *INVAN6* expression in roots; (**M**) *INVAN7* expression in leaves; (**N**) *INVAN7* expression in roots; (**O**) *INVAN8* expression in leaves; (**P**) *INVAN8* expression in roots; (**Q**) *INVVR1* expression in leaves; (**R**) *INVVR1* expression in roots; (**S**) *INVVR2* expression in leaves; (**T**) *INVVR2* expression in roots; (**U**) *INVCW5* expression in leaves; (**V**) *INVCW5* expression in roots; (**W**) *INVCW6* expression in leaves; (**X**) *INVCW6* expression in roots. Actin, TIP41 and 17sRNA were used as internal controls. Data are means ± SD (*n* = 3). Different letters above bars indicate a significant difference at *p* < 0.05. CT, control. UD, undetected. *** *p* < 0.001.

The changes in the expression of all tomato A/N invertase and vacuolar acid invertase genes as well as two major cell wall invertase genes were investigated under the different treatment conditions (Figure 10A–X). The results showed that the expression of *INVAN1* in the leaf was significantly decreased by NaCl, H_2_O_2_, ABA and SA treatments at early stages, and it was recovered to the control level or even increased after 72 h of treatment (Figure 10A). The expression of *INVAN2* and *INVAN3* was increased by PEG treatment in the leaf and root (Figure 10C–F). In the leaf, NaCl, H_2_O_2_, ABA and SA treatments all decreased the *INVAN3* expression at 12 and 24 h, but the expression was unaltered or increased at 72 h (Figure 10E), while in the root, the *INVAN3* expression was stimulated by salt stress (Figure 10F). Salt stress also promoted the expression of *INVAN4* in the leaf (Figure 10G). The expression of *INVAN5* was inhibited in both leaves and roots under all treatment conditions (Figure 10I,J). The expression of *INVAN6* and *INVAN8* was stimulated in the root by PEG and NaCl treatments at 12 h and they were also increased in the leaf by ABA treatment at 72 h (Figure 10K,L,O,P). The *INVAN7* expression was decreased by different chemical treatments, especially in the root (Figure 10M,N). In the leaf, the expression of *INVVR1* was promoted only by PEG, H_2_O_2_, ABA at 72 h, while in the root, the expression was stimulated by PEG, NaCl and ABA treatments. All the treatments inhibited the expression of *INVVR2* and *INVCW6* in the leaf and root as well as the *INVCW5* expression in the leaf at early stages (Figure 10S,T).

## 3. Discussion

SPS, SUS and INV are the main enzymes that regulate sugar metabolism. With the development of sequencing technology and bioinformatics, the family genes encoding these enzymes have been identified and the structure characteristics have also been analyzed in some plants, such as Arabidopsis and rice [6,7,11,14]. In tomato, although some genes of *SPS*, *SUS* and *INV* families have been identified [2,5,11,17], a comprehensive analysis of the structure characteristics and evolution of these genes is still lacking. Moreover, the expression responses of these genes to environmental stimuli are largely unknown. In this study, comprehensive analyses on the gene characteristics and evolution of *SPS*, *SUS* and *INV* in tomato were conducted, and the expression responses of these genes to different stress stimuli were also investigated.

### 3.1. Evolution and Structure of SPS Genes

SPS is the key enzyme responsible for sucrose synthesis [1]. The *SPS* gene family in plants is relatively small: there are 3–5 *SPS* genes in most species [50]. In this study, four SPS genes were identified in tomato (Table 1), which is consistent with most plants. The relatively small *SPS* family in plants may be partly related to the lack of duplication events of this gene, at least in tomato, as observed here (Figure 3A). In this study, synteny analysis demonstrated that *AtSPS1F*-*SlSPS1*, *SlSPS1*-LOC_Os06g43630 and *AtSPS3F-SlSPS3* were syntenic gene pairs, respectively (Figure 3B), implying the interspecific conservation of these genes.

The identified four SlSPS members together with the SPS in Arabidopsis and rice were categorized into four families (Figure 1A). SlSPS1 and SlSPS2 were grouped into family A, while SlSPS3 and SlSPS4 belonged to families B and C, respectively (Figure 1A). The presence of family D SPS in rice but absence in tomato and Arabidopsis (Figure 1A) is consistent with the finding of Castleden et al. [8], who suggested that families A, B and C of SPS widely exist in monocotyledons and dicotyledons, whereas family D SPS only exist in the *Poaceae* (monocotyledons). In addition, the existence of families B and C SPS in both monocotyledon (rice) and dicotyledons (Arabidopsis and tomato) implies that the differentiation of SPS families was completed before the differentiation of monocotyledons and dicotyledons. It has been suggested that the SPS family differentiation event occurred about 200 million years ago [51].

Analysis of intron-exon structure is helpful to study the possible origin and relationship of genes [11]. Here, in tomato, the four *SlSPS* genes had similar exon-intron structures, and the numbers of both exons and introns were the same in *SlSPS1* and *SlSPS2* (Table 1; Figure S1). Compared with *SlSPS1* and *SlSPS2*, *SlSPS3* and *SlSPS4* experienced intron loss or gain events. *SlSPS3* had a longer first exon due to the loss of the equivalence of first intron, while the equivalence of the fifth exon of *SlSPS4* was inserted by the 238 bp intron, resulting in an additional exon compared with *SlSPS1* and *SlSPS2* (Figure S1A). Similar intron loss and gain events were also observed in litchi [50], reflecting the evolutionary relationship of these individual *SPS* genes plants.

All the four SPS members had glycosyl transferase and S6PP domains (Figure 2A), which are unique to SPS family [47]. Ten conserved motifs were predicted in each SPS protein, and most of the motifs were repeated once (Figure 2A and Figure S3A). It is interesting to note that motif 6, which belongs to the GTB-type-superfamily, was repeated at the N-terminal of SlSPS2 (Figure 2A). The functional significance of this additional repeat remains to be investigated.

### 3.2. Evolution and Structure of SUS Genes

In plants, the number of *SUS* gene members varies greatly among species. For instance, only two *SUS* genes were identified in *Amborella trichopoda* [11], whereas in Chinese pear, thirty *SUS* genes were reported [13]. The difference in family member numbers may be related to the difference in family gene expansion [52]. In this study, six *SUS* genes were identified in tomato (Table 1). *SlSUS3* and *SlSUS5* were identified as tandem duplication genes, while *SlSUS1* and *SlSUS5* were identified as segmental duplication gene pairs (Figure 4A). Therefore, the *SUS* duplication events have contributed to the expansion of this gene family.

In plants, SUS can be classified into three subfamilies—SUS I, SUS II and SUS III [1]. Phylogenetic analysis of SlSUS together with the SUS in Arabidopsis and rice demonstrated that in each subfamily, there were SUS from both monocotyledonous (rice) and dicotyledonous (Arabidopsis and tomato) plants (Figure 1B). This suggests that the subfamily differentiation of SUS happened before the split of monocotyledons and dicotyledons.

A previous study has revealed that *SUS* genes were highly conservative in many dicotyledons and monocotyledons [11]. Consistent with this, our study shows that the exon-intron structure and arrangement of the *SUS* family genes in tomato were also generally conservative (Figure S1B and Figure 2B). However, there were still some differences in the exon-intron structure. For instance, the equivalences of the 5th and 12th introns in *SlSUS4* were lost in all the *SUS I* genes (*SlSUS1*, *SlSUS3* and *SlSUS5*), while two *SUS III* genes (*SlSUS6* and *SlSUS7*) lost the equivalence of the 12th but not the 5th intron (Figure S1B). Intriguingly, compared with other subfamily members, two *SUS III* subfamily genes, *SlSUS6* and *SlSUS7*, respectively, had one and two more exons at the 3′ end (Figure S1B), and the corresponding amino acid sequences were longer (Table 1). In addition, it is noted that *SlSUS1* and *SlSUS3*, respectively, had a longer UTR in the 5′ end. The significance of the differential intron loss, 3′ end extension and different UTR length still remains to be explored in the future.

### 3.3. Evolution, Structure and Classification of INV Genes

The number of *INV* family genes also varies greatly among species. There were 14 *INV* genes in sugarcane [15], while this number in soybean is 32 [16]. In the present study, nineteen *INV* genes were identified in tomato, including eleven acid invertase genes and eight A/N invertase genes (Table 1). The interspecific variation in the number of *INV* genes should be associated with the difference in gene duplication events as found in this study—there were three tandem duplication pairs (*INVCW3*/*INVCW4*, *INVCW5*/*INVCW6* and *INVCW8*/*INVCW9*) and two segmental duplication gene pairs (*INVCW3*/*INVCW5*, *INVAN6*/*INVAN8)* in tomato (Figure 5A). In addition, ten syntenic gene pairs between tomato and Arabidopsis and nine pairs between tomato and rice were identified (Figure 5B), suggesting the evolutionary conservation of *INV* genes in plants.

In this study, eleven acid invertase genes including nine cell wall invertase genes and two vacuolar invertase genes were identified (Table 1). Most *SlINVCW* genes had six exons, except *SlINVCW2*, which had eight exons, while the *SlINVVR* genes contained seven exons (Table 1; Figure S1C). Compared with most *SlINVCW* genes, *SlINVCW2* contained two introns while the *SlINVVR* genes had one intron in the equivalence of the third exon (Figure S1C). Phylogenetic analysis indicated that *SlINVCW1/2* and *SlINVVR* genes evolved earlier than other *SlINVCW* genes (Figure 1C and Figure 2C). This suggests that the acid invertase genes might have experienced intron losses during evolution. The second exon of all the 11 identified *INV* genes was a 9 bp long mini exon, which encoded three amino acids (Figure 2C and Figure S1C). Such a mini exon is a typical structure characteristic of acid invertase genes in plants [53], and it is also the smallest exon that has even been found in plants [54]. It is noted that the first exon was longer in *SlINVVR* genes than *SlINVCW* genes (Figure S1C). The DUF3357 domain was encoded by the first exon, and it was present in SlINVVRs but absent in SlINVCWs, resulting in longer N-terminal of the former (Figure 2C).

Eight A/N invertase genes were identified in tomato (Table 1). According to the sequence homology, these invertases could be divided into two group: the α group (SlINVAN1, 2, 6 and 8) and β group (SlINVAN3, 4, 5 and 7) (Figure 1D). The two groups had different exon numbers: 6–7 exons in the α group and four in the β group (Figure S1). Our results are generally consistent with previous findings that the α group and β group genes typically have six and four exons, respectively [55,56]. However, there was an exception in *SlINVAN1*, which contained seven exons (Figure S1D). It should be pointed out that the exon numbers of *SlINVAN1* and *SlINVAN6* found in this study were inconsistent with the study of Pan et al. [2]. The differences may be related to the differences in the genomic annotations, which remain to be further explored. The two groups of tomato invertases also differed in the subcellular localization and the length of amino acid sequence. In this study, the α group invertases were localized either in mitochondria (SlINVAN1 and 2) or cytoplasm (excluding cytosol, SlINVAN6 and 8), whereas the β group (SlINVAN3, 4, 5 and 7) were localized in the cytosol (Table 1). The α group proteins had 653–672 amino acids, whereas the β group contained 551–570 amino acids (Table 1). The longer protein sequence in the α group was due to the longer N-terminal region (Figure 2D). Whether there were more signal peptide sequences in the long N-terminal region and, if there were, whether they were related to the multilocalization of the α group invertases (Table 1) remains to be investigated.

### 3.4. Tissue Expression Pattern of SPS, SUS and INV Genes

Comprehensive expression analysis of all gene family members may help understand their functions. In tomato, the expression profiles of *SPS*, *SUS* and *INV* genes have not been comprehensively analyzed in different tissues, except those of SUS and part of INV genes, but on in a few tissues [2,11]. In this study, in silico analysis on the expression of *SPS*, *SUS* and *INV* genes was conducted in 19 tissues of tomato, and the results showed that there were expression differences among family members and tissues (Figure 6A–C).

Among the four *SlSPS* genes, *SlSPS1* demonstrated relatively higher expression in different tissues except pollen (Figure 6A). In the leaves, the expression of *SlSPS1* and *SlSPS3* was higher than that of *SlSPS2* and *SlSPS4*, while in the root, the expression of *SlSPS1* was the highest among the family members (Figure 6A). These results were confirmed by quantitative PCR in ‘Alisa Craig’ (Figure 7A). Bahaji et al. observed that, compared with the wild type, Arabidopsis mutants *spsa1/spsc* and *spsa1/spsa2/spsc* had smaller rosettes [18], flowers and siliques; moreover, the seeds of *spsa1/spsb/spsc* and *spsa1/spsa2/spsb/spsc* mutants germinated poorer and the plants were sterile. Therefore, the highly expressed *SlSPS*, especially *SlSPS1* in different tissues, may play key roles in tomato growth and development, such as seed germination, vegetative growth, flower and fruit development.

In this study, it is noted that *SlSUS1* demonstrated the highest expression in fruits, and the expression of *SlSUS3* was also relatively high (Figure 6B). In the fruit, both *SlSUS1* and *SlSUS3* had the highest expression in the outer pericarp (Figure 6B). D’Aoust et al. reported that antisense inhibition of TOMSSF (*SlSUS1* in this study; Table 1) reduced sucrose import into the tomato fruit and decreased fruit setting [40]. Zhao et al. found that downregulating the expression of *FaSS1*, a *SUS* gene in strawberry, delayed fruit ripening [30]. These studies suggest that *SlSUS1* and *SlSUS3* may play important roles in tomato fruit development. Among the six *SlSUS* genes, both *SlSUS3* and *SlSUS5* had the highest expression in the root (Figure 6B and Figure 7B), indicating that these two genes mainly contributed to the strength of this sink tissue. In addition, in the vascular tissue and leaf, *SlSUS3* had a higher expression (Figure 6B). The highly expressed *SlSUS3* gene in vascular tissues may be involved in the synthesis of both cellulose and callose, as suggested previously in Arabidopsis [26] and poplar [27].

Among the cell wall-localized acid invertase genes, *SlINVCW3* demonstrated relatively high expression in different tissues, especially vascular tissue, flower and fruit (Figure 6C). These results are consistent with previous studies [43,57]. *SlINVCW3* (named *LIN5* in previous studies, Table 1) has been suggested to function in ovary-to-fruit transition in the sieve elements by generating a glucose signal, and thus regulating cell division [57]. Silencing this gene not only affected the fertility and fruit development, but also influenced the fruit hormone level [33]. These studies suggest that *SlINVCW3* plays an important role in the reproductive growth of tomato plants. In this study, it was observed that *SlINVCW4* was highly expressed in pollen (Figure 6C). The high expression of this gene has been confirmed to be required for pollen development [58]. It is interesting to note that *SlINVVR1* was highly expressed in different tissues, especially in parenchyma and fruit (Figure 6C). Qin et al. reported that the inhibitor of *VI* (*SlINVVR1* in this study) could mediate sucrose metabolism and affect fruit ripening, suggesting the role of *SlINVVR1* in these processes [17]. The function of the highly expressed *SlINVVR1* in parenchyma remains to be investigated. Among the eight *SlINVAN* genes, *SlINVAN4* demonstrated relatively higher expression in different tissues (Figure 6C). Recently, Leskow et al. found that silencing this gene impaired the growth phenotype, delayed flowering and reduced fruit setting [45]. This suggests that the high expression of this gene is required for the normal growth, flowering and fruit development. *SlINVAN6* had the highest expression in pistil (Figure 6C), and its role remains to be investigated.

In a word, the highly expressed genes, including *SlSPS1*, *SlSUS1*, *SlSUS3*, *SlINVCW3*, *SlINVVR1* and *SlINVAN4* in most parts of the fruit (Figure 6A–C), may be involved in the so called ‘futile cycles’ of sucrose [59], which regulate sugar accumulation and/or sugar signaling, and thus fruit development. Despite the high expression in fruits, these genes also demonstrated differential expressions in different parts of the fruit (Figure 6A–C). Therefore, the exact regulatory functions of these genes in sugar accumulation in fruits remain to be further investigated. Although the functions of some members of these gene families have been clarified in flowers and fruits, those of other members, especially the *SlINVAN* genes, are mostly unclear. Moreover, the genes of these three families may play important roles in other physiological processes except flower and fruit development. For instance, it has been shown that *SlSUS* participates in the regulation of early leaf morphology development [28]. However, relevant information in these aspects is still limited, and more work is needed in the future.

### 3.5. Expressions of SPS, SUS and INV Genes under Stresses

Although the roles of some (not all) *SPS*, *SUS* and *INV* genes in flower and fruit development of tomato have been investigated, the responses of these genes to environmental stimuli and their roles in stress tolerance still remain largely unknown. In this study, different chemicals were applied to tomato seedlings to simulate various stress conditions, and the expression responses of the *SPS*, *SUS* and *INV* family genes were investigated. In general, we observed that within each gene family, the members demonstrated differential expression responses to environmental stimuli and in different tissues (Figure 8, Figure 9 and Figure 10). It is interesting to see that some genes demonstrated consistent expression responses to different treatments in the leaves and/or roots. For example, the *SlSPS3* expression in leaves was downregulated and the *SlSPS1* expression in roots was upregulated under all treatment conditions (Figure 8B,E). Under PEG, NaCl, H_2_O_2_ and ABA treatments, the expression of *SlSUS1*, *SlSUS3* and *SlSUS4* was mostly increased in both leaves and roots. In addition, both *SlINVAN5* and *SlINVAN7* demonstrated downregulated expression under the five treatment conditions (Figure 10I,J,M,N). These results suggest that *SlSPS1*, *SlSPS3*, *SlSUS1*, *SlSUS3*, *SlSUS4*, *SlINVAN5* and *SlINVAN7* may be the major genes responding to the different exogenous stimuli in tomato. It is also noted that the expression of some genes was not altered or changed much by the treatments, such as *SlSPS2* and *SlINVAN6* in leaves under NaCl treatment, *SlINVAN4* in roots under NaCl stress, *SlSUS4* and *SlINVAN6* in roots, and *SlSUS6*, *SlSUS7* and *SlINVAN2* in both leaves and roots under SA treatment. The differential expression responses of these genes to environmental stresses have been reported previously. For instance, Hu et al. observed that in perennial ryegrass, the expression of *SPS* was slightly increased in the roots but decreased in the stem under salt stress, while in the leaves, the expression was increased in the salt-sensitive accession but remained unchanged in the tolerant accession [60]. Solís-Guzmán et al. reported that the expression of *AtSPS2F* and *AtSPS4F* were up-regulated under osmotic stress, whereas those of *AtSPS1F* and *AtSPS3F* were down-regulated [61]. Yang et al. found that in apple leaves, drought stress increased the expression of *SPS2*, *SPS3*, *SPS4*, *SUSY1*, *SUSY3*, *SUSY5*, *NINV1* and *NINV2*, but decreased the expression of *SPS1*, *SUSY2*, *CWINV1*, *CWINV2*, *NINV3* and *AINV1*. They also observed that the expression changes in *SPS5*, *SPS6*, *SUSY4* and *AINV3* expression were dependent on stress duration—increased in the early stress stage but decreased in the later stage [3]. Dahro et al. reported that *PtrA/NINV* expression in *Poncirus trifoliata* was upregulated by low temperature, salt, dehydration, sucrose and ABA, but downregulated by glucose. These studies suggest that each isoform of these gene families may have distinct functions under different environmental stimuli and in different tissues [62].

It is noticed that under the exogenous treatments, the changes in the expression of *SlSUS* genes were different from those of *SlINV* genes. In most cases, the expression of *SlSUS* genes was upregulated by different treatments (Figure 9), and the upregulation magnitudes were higher than those of *SlINV* genes (Figure S6). This seems to suggest that the *SlSUS* genes may play more important roles than the *SlINV* genes in the response to environmental stimuli. In future, more work is needed to investigate the roles of individual genes of the three families in stress tolerance in tomato plants.

## 4. Materials and Methods

### 4.1. Identification of SPS, SUS and INV Genes in Tomato

To identify the *SPS* genes in tomato (*Solanum lycopersicum*), the annotated tomato genome was downloaded from the website of National Centre for Biotechnology Information (http://www.ncbi.nlm.nih.gov, accessed on 26 February 2020). The sequences of all known AtSPS proteins in Arabidopsis (At5g20280 (AtSPS1F), At5g11110 (AtSPS2F), At1g04920 (AtSPS3F) and At4g10120 (AtSPS4F)) were used as queries to search the tomato genome by BLASTp. Subsequently, the candidate sequences were subjected to the second round BLASTp against SWISS-PROT database to check whether the corresponding sequences were close to the identified SPS family in other plants. Batch-CD was then employed to screen whether the candidate proteins had the glucosyl transferase glycos-transf-1 domain and S6PP domain that are unique to sucrose phosphate synthase family, and partial and defective sequences were eliminated during manual verification. When there were multiple transcripts for a gene, the longest variant was selected. The sequence length, molecular weight, and isoelectric point information of the identified SPS proteins were obtained using the online tool—ExPASy (https://web.expasy.org/protparam, accessed on 10 March 2020). The online tool PLANT-PLOC (http://www.csbio.sjtu.edu.cn/bioinf/plant, accessed on 12 March 2020) was employed to predict the protein subcellular localizations [63], and Kinasephos (http://kinasephos.mbc.nctu.edu.tw, accessed on 15 March 2020) was used to predict the phosphorylation sites.

Identification of SUS and INV genes in tomato was as described for the identification of SPS genes. The query sequences for SUS protein identification were from Arabidopsis (At5g20830 (AtSUS1), At5g49410 (AtSUS2), At4g02280 (AtSUS3), At3g43190 (AtSUS4), At5g37180 (AtSUS5) and At1g73370 (AtSUS6)). The accessions of query proteins for INV identification were At3g52600 (AtCWINV2), At2g36190 (AtCWINV4), At3g13784 (AtCWINV5), At5g11920 (AtCWINV6), At1g55120 (AtFRUCT5), At3g13790 (AtBFRUCT1), At1g62660, At1g12240 (AtATV12), At1g56560 (AtAN-INVA), At4g34860 (AtAN-INVB), At3g06500 (AtAN-INVC), At1g35580 (AtAN-INVG), At4g09510 (AtAN-INV1), At1g22650 (AtAN-INVD), At1g72000 (AtAN-INVF), At5g22510 (AtAN-INVE), At3g05820 (AtAN-INVH). For SUS identification, the candidate sequences were screened with Batch-CDD online tool to see if they share the sucrose synthase and glucosyl-transferase domains that are unique to sucrose synthase [64]. For INV identification, the sequences that contain a glyco-hydro-32N, glyco-hydro-32C domain, a cysteine catalytic domain MWECP/V and a β-furosidase motif NDPNG/A were identified as acid invertase, whereas if the candidate sequences only contain a glyco-hydro-100 domain, they were identified as alkaline/neutral invertases [54].

### 4.2. Phylogenetic Relationship, Gene Structure and Protein Motif Analysis

A phylogenetic tree was constructed by MEGA-X using the maximum likelihood method with Poisson model and 1000 bootstrap replications [65]. SPS, SUS and INV protein sequences of Arabidopsis and rice were downloaded from UniProt website (https://sparql.uniprot.org, accessed on 2 March 2020). The gene structures of SlSPS, SlSUS and SlINV were analyzed by gene structure display server (GSDS) online program (http://gsds.cbi.pku.edu.cn, accessed on 7 April 2020). The NCBI BATCH CD-search tool (https://www.ncbi.nlm.nih.gov/Structure/bwrpsb/bwrpsb.cgi, accessed on 12 April 2020) was used to analyze the conserved domain structure based on the corresponding protein sequence, and Pfam database was chosen for searching. The conserved motifs were predicted by the MEME Suite tools (http://meme-suite.org, accessed on 12 April 2020) [66], and the number of motif parameters was limited to less than 10 manually.

### 4.3. Chromosome Distribution, Gene Duplication and Syntenic Relationship Analysis

The physical location information of *SlSPS*, *SlSUS* and *SlINV* genes was obtained from the GCF_000188115.4_SL3.0_genomic database in NCBI, and all the identified genes were mapped to the tomato chromosomes by TBtools V1.089 [67]. Multiple collinear scanning toolkits (MCScanX) with default parameters were used to analyze the gene duplication events [68]. The syntenic relationship of genes in tomato, Arabidopsis and rice was analyzed using Dual Synteny Plotter software [67].

### 4.4. Plant Preparation and Chemical Treatment

Tomato (‘Alisa Craig’) seeds were sterilized in warm water at 55 °C for 15 min, and then washed with sterile water at room temperature. The sterilized seeds were placed on two layers of filter paper in a petri dish and cultured in darkness at 30 °C for 2 d. The germinated seeds were sown in plugs and seedlings at two-leaf stage were transplanted into dark plastic boxes containing 1/4 Hoagland nutrient solution (pH 5.8) [69]. Seven days later, when the seedlings had three fully expanded leaves, the culture medium was replaced with 1/2 Hoagland solution (pH 5.8), in the absence or presence of 20% (*w*/*w*) polyethylene glycerol 6000 (PEG-6000), 150 mM NaCl, 1.5 mM H_2_O_2_, 50 μM abscisic acid (ABA) or 200 μM salicylic acid (SA). Seedlings cultured in 1/2 Hoagland solution were employed as control. After 12, 24 and 72 h of treatment, all the roots and leaves were separately collected, immediately frozen in liquid nitrogen and then stored at −80 °C for analysis of gene expression.

### 4.5. RNA Extraction and Quantitative RT-PCR Analysis

Total RNA of leaves or roots was extracted with an E.Z.N.A. Plant RNA Kit (Omega, GA, USA). Genomic DNA in the RNA samples was erased by both adsorption column in the E.Z.N.A plant RNA Kit (Omega) and DNase in HiScript^®^ II Q RT SuperMix for qPCR (+gDNA wiper, Vazyme, Nanjing, China). The first strand of cDNA was synthesized with a HiScript^®^ II Q RT SuperMix for qPCR. Quantitative RT-PCR was performed on a Real-Time PCR system (QuantStudioTM 5, Applied Biosystems, Waltham, MA, USA) using AceQ^®^ qPCR SYBR Green Master Mix Kit (Vazyme). Actin, TIP41 and 17sRNA were employed as internal controls [42,70]. All the primers used were listed in Table S1. The relative expression levels of *SlSUS*, *SlINV* and *SlSPS* genes were calculated using the 2^−∆∆Ct^ method [71].

### 4.6. Tissue Expression Characteristics of SPSs, SUSs and INVs in Tomato

The tissue expression patterns of *SPS*, *SUS* and *INV* genes in tomato were determined bioinformatically and experimentally. The expression values in different tissues were retrieved from large scales of RNA-Seq with Genevestigator (https://genevestigator.com, accessed on 11 May 2020), a software that provides the average signal intensity values of a gene from a high diversity of experiments covering different tissues, ages, and treatments [72,73]. The IDs of all *SPS*, *SUS* or *INV* genes were given as inputs in the Anatomy tool of Genevestigator and searched against the RNA-seq database of *Solanum lycopersicum* involving seed, roots, shoot apex, leaf, vascular tissue, parenchyma, floral meristem, flower, pollen, pistil, ovule, ovary wall, fruit, outer pericarp, pericarp, inner pericarp (columella), locular tissue, placenta and septum. The source of RNA-seq data are provided in Table S2. The expression *SlSUSs*, *SlINVs* and *SlSPSs* in leaves and roots was also analyzed by quantitative RT-PCR.

### 4.7. Statistical Analysis

The data were subjected to one-way analysis of variance with Duncan’s multiple range test at *p* < 0.05 using SPSS 18.0 (IBM, Armonk, NY, USA).

## 5. Conclusions

Four *SPS* genes, six *SUS* genes and nineteen *INV* genes were identified in tomato. Tandem and segmental duplications contributed to the expansion of *SlSUS* and *SlINV* families, and no gene duplication was found in the *SlSPS* family. SlSPS could be classified into three families (A, B and C), and SlSUS could be classified into three subfamilies (I, II and III). The *SlINV* genes included eleven acid invertase genes and eight A/N invertase genes. The differentiation of *SlSPS* and *SlSUS* might have completed the differentiation of monocotyledons and dicotyledons. The conserved motifs were mostly consistent within each protein family or subfamily. *SlSPS*, *SlSUS* and *SlINV* genes showed differential expressions among family members and tissues, and in response to different environmental stimuli. *SlSPS1*, *SlSPS3*, *SlSUS1*, *SlSUS3*, *SlSUS4*, *SlINVAN5* and *SlINVAN7* showed consistent expression responses to different treatments in the leaves and/or roots, and thus may be the major genes responding to exogenous stimuli. The results suggest that the individual isoforms of these gene families may demonstrate differential functions under environmental stimuli and in different tissues. The study not only provides the structure characteristics and evolution information of genes encoding sucrose metabolism related enzymes, but also lays a foundation for investigating the functions of individual isoforms of each enzyme in plant growth, development and stress tolerance.

## Figures and Tables

**Table 1:** Gene structure and protein characteristics of SUSs, INVs and SPSs in tomato.

Gene			Gene Structure				Protein Characteristics					Gene Name in the Literature
Name	Locus	Chr	Sequence Length (bp)	CDS Length (bp)	Exon No.	Intron No.	Length (Aa)	IP	MW (KDa)	Subcellular Localization	Phosphorylation Site No.	(References)
*SPS1*	Solyc07g007790	7	8511	3165	13	12	1054	6.05	118.5	PM	24	*SPSA1* [17]
*SPS2*	Solyc08g042000	8	7283	3147	13	12	1048	6.26	117.9	PM	21	*SPSA2* [17]
*SPS3*	Solyc09g092130	9	8270	3195	12	11	1064	6.13	119.6	PM	15	*SPSB* [17]
*SPS4*	Solyc11g045110	11	7534	3090	14	13	1029	6.59	116.2	PM	21	*SPSC* [17]
*SUS1*	Solyc12g009300	12	5834	2418	13	12	805	5.94	92.5	PM	9	*SUS1* [39]; *TOMMSSF* [40]
*SUS3*	Solyc07g042550	7	5663	2418	13	12	805	5.96	92.5	PM	13	*SUS3* [41]
*SUS4*	Solyc09g098590	9	6464	2439	15	14	812	5.91	92.9	PM	18	*SUS4* [42]
*SUS5*	Solyc07g042520	7	3624	2412	11	10	803	5.97	91.6	PM	13	*SS5* [17]
*SUS6*	Solyc03g098290	3	4379	2676	15	14	891	5.87	100.7	PM	20	*SS6* [17]
*SUS7*	Solyc02g081300	2	4757	2550	14	13	849	6.94	96.3	PM	14	*SS7* [17]
*INVCW1*	Solyc03g121680	3	2716	1716	6	5	571	6.8	64.2	CW	13	
*INVCW2*	Solyc06g064620	6	2834	1596	8	7	531	4.96	59.7	CW	8	
*INVCW3*	Solyc09g010080	9	3863	1755	6	5	584	9.2	67.2	CW *	8	*LIN5* [43]
*INVCW4*	Solyc09g010090	9	3530	1752	6	5	583	6.93	66.2	CW	14	*LIN7* [43]
*INVCW5*	Solyc10g083290	10	5513	1749	6	5	582	9.23	65.9	CW	9	*LIN6* [43]
*INVCW6*	Solyc10g083300	10	4528	1560	6	5	519	8.69	58.8	CW	8	*LIN8* [43]
*INVCW7*	Solyc10g085360	10	4459	1737	6	5	578	8.7	65.9	CW	5	
*INVCW8*	Solyc10g085640	10	3498	1731	6	5	576	7.26	65.7	CW	5	
*INVCW9*	Solyc10g085650	10	3659	1737	6	5	578	6.39	66.6	CW	10	
*INVVR1*	Solyc03g083910	3	4187	1911	7	6	636	5.54	70.1	Vac	8	*VI* [44]
*INVVR2*	Solyc08g079080	8	4194	1959	7	6	652	6.21	72.8	Vac	9	*LIN9* [17]
*INVAN1*	Solyc01g058010	1	5694	2010	7	6	669	5.84	76.0	Mito	10	*NI1* [17]
*INVAN2*	Solyc01g100810	1	5852	1962	6	5	653	8.18	74.5	Mito	13	*NI2* [4,17]
*INVAN3*	Solyc01g111100	1	5189	1707	4	3	568	6.45	64.9	Cyt	13	*CIN2* [4]
*INVAN4*	Solyc04g081440	4	4713	1713	4	3	570	5.97	65.2	Cytosol *	13	*CIN3* [4]; *NI6* [45]
*INVAN5*	Solyc06g065210	6	5788	1656	4	3	551	6.16	62.8	Cyt	17	*NI3* [17]; *CIN4* [4]
*INVAN6*	Solyc11g007270	11	6087	1968	6	5	655	5.84	73.5	Chl	12	*CIN5* [4]
*INVAN7*	Solyc11g020610	11	6175	1659	4	3	552	6.06	62.6	Cyt	16	*NI4* [17]; *CIN6* [4]
*INVAN8*	Solyc11g067050	11	4291	2019	6	5	672	6.89	76.2	Chl	8	*NI5* [17]; *CIN7* [4]

* Verified experimentally. Abbreviations: Aa, amino acid; CDS, coding DNA sequence; Chl, chloroplast; Chr, chromosome; CW, cell wall; Cyt, cytoplasmic; IP, isoelectric point; Mito, mitochondrial; MW, molecular weight; PM, plasma membrane; Vac, vacuole.

## Data Availability

The data presented in this study are available on request from the corresponding author.

## Supplementary Materials

The following are available online at https://www.mdpi.com/article/10.3390/ijms22094698/s1.
