# Supplementary figures and images for: Structure and Expression Analysis of Sucrose Phosphate Synthase, Sucrose Synthase and Invertase Gene Families in Solanum lycopersicum
Source: Int J Mol Sci. 2021 Apr 29;22(9):4698. doi: 10.3390/ijms22094698 (PMC8124378; doi:10.3390/ijms22094698)

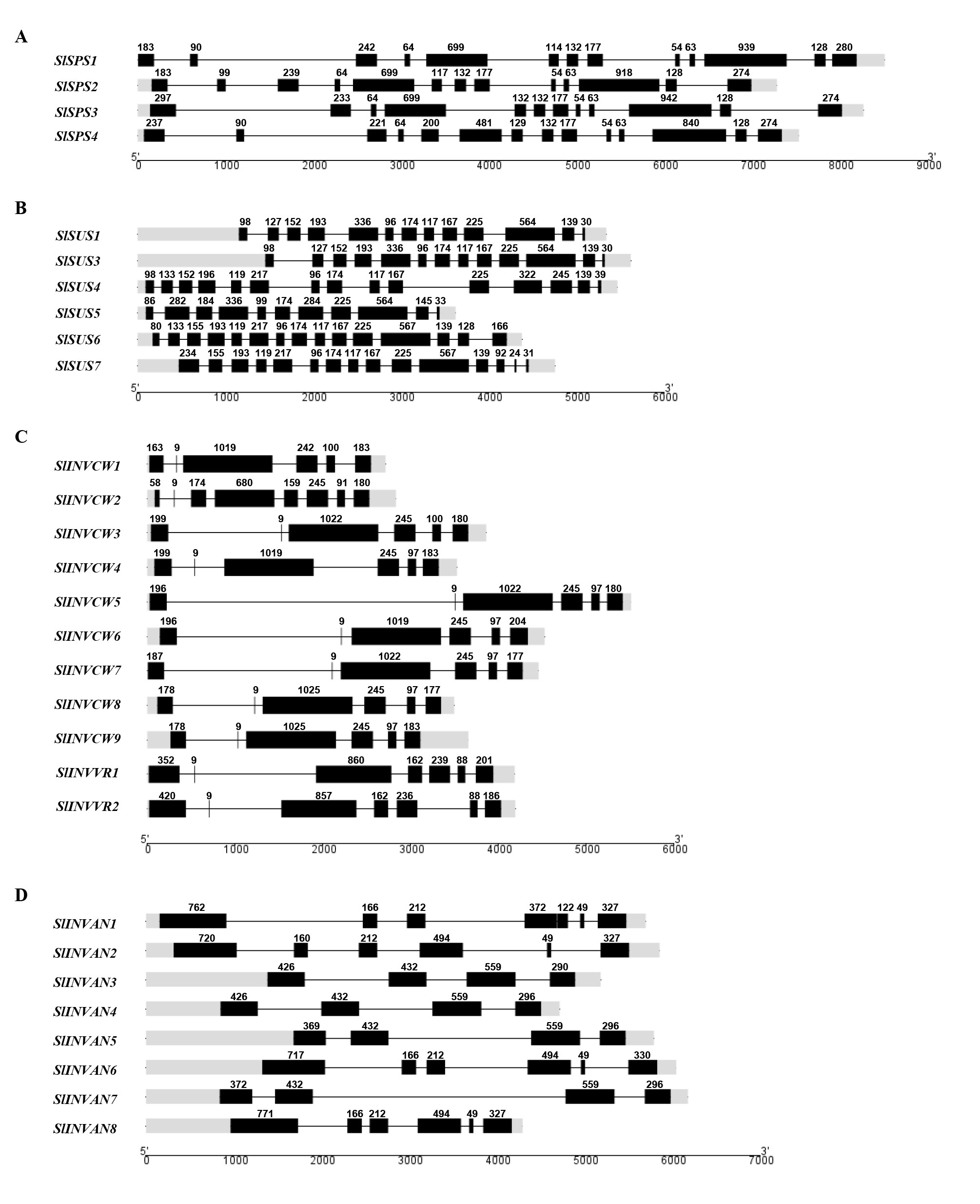

Supplement: Supplementary file 1 [file ijms-22-04698-s001.zip › Fig S1 Exon-intron structure of SPS, SUS and INV.jpg]

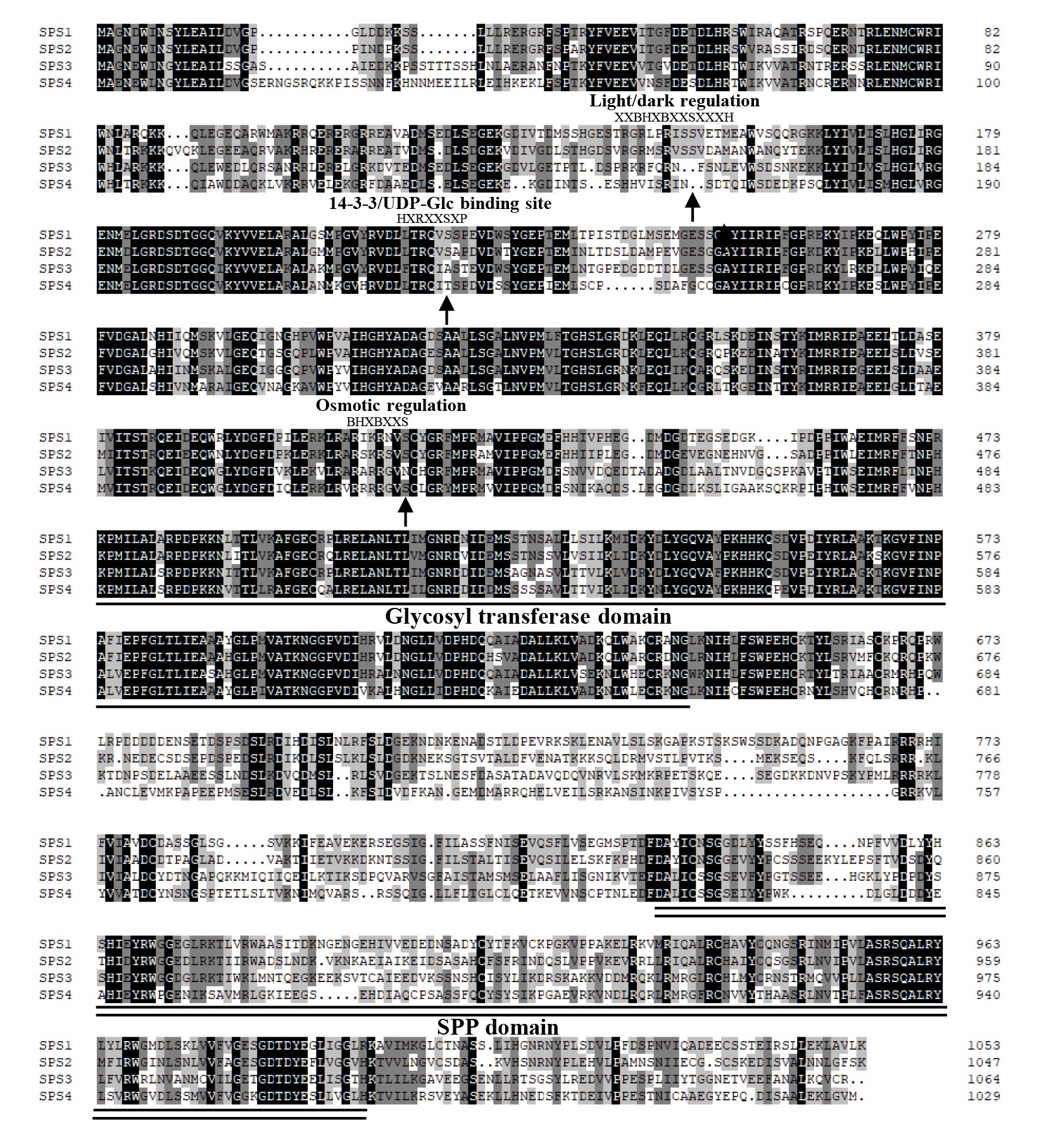

Supplement: Supplementary file 1 [file ijms-22-04698-s001.zip › Fig S2 SlSPS domain.jpg]

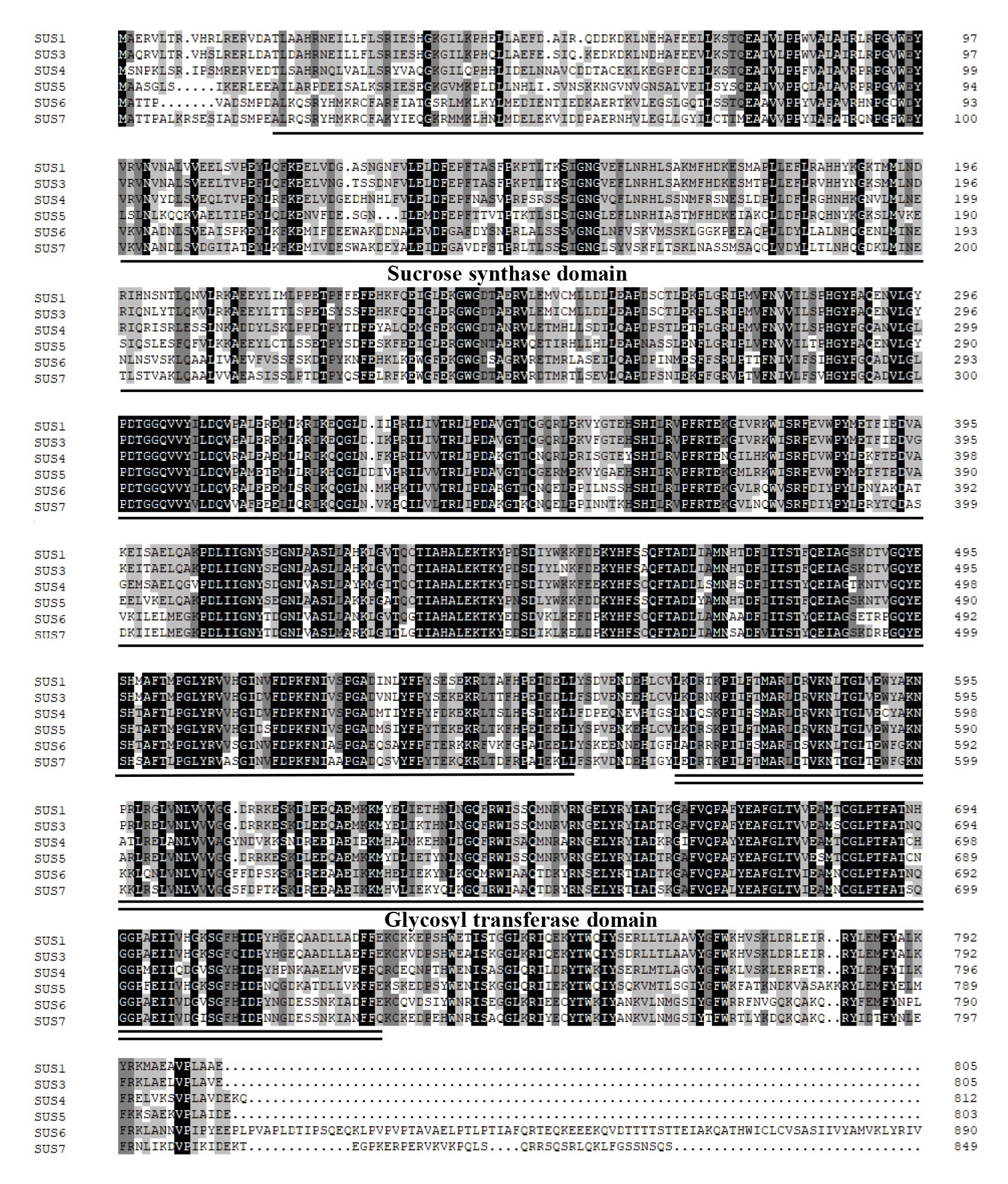

Supplement: Supplementary file 1 [file ijms-22-04698-s001.zip › Fig S4 SlSUS domain.jpg]

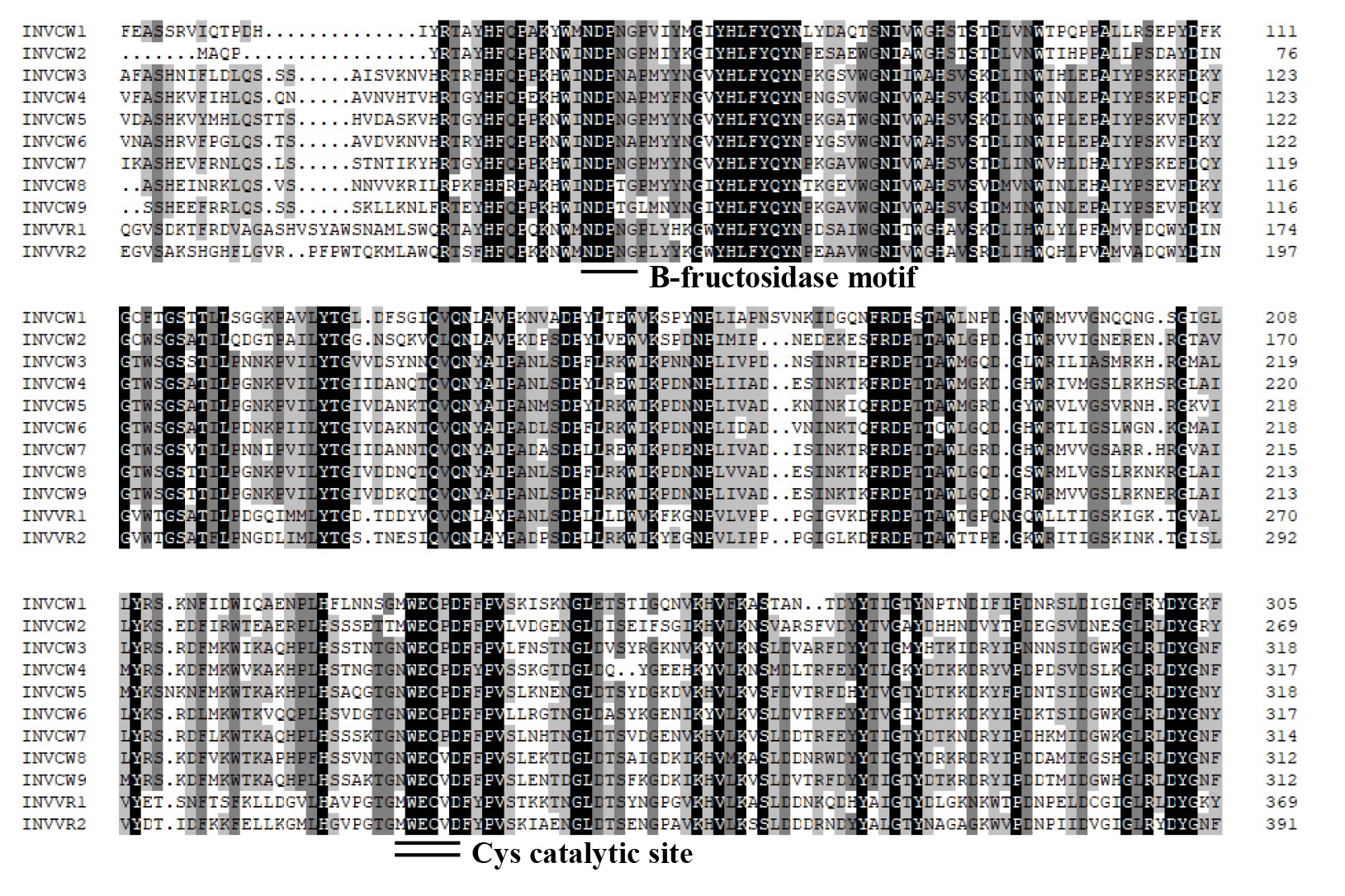

Supplement: Supplementary file 1 [file ijms-22-04698-s001.zip › Fig S5 SlINV domain.jpg]

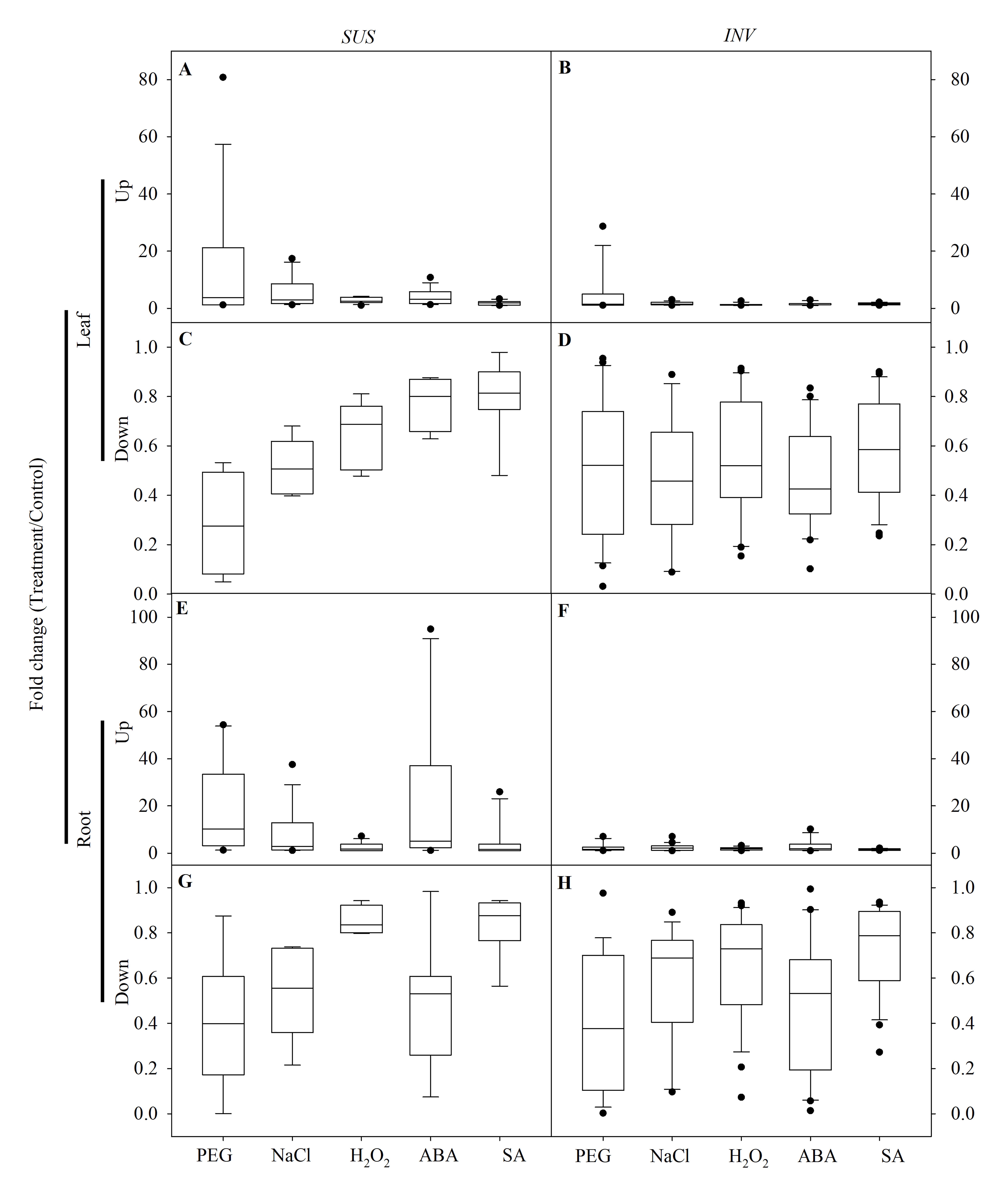

Supplement: Supplementary file 1 [file ijms-22-04698-s001.zip › Fig S6 Fold change of SUS & INV.jpg]
